# Closure strategy for endoscopic pituitary surgery: Experience from 3015 patients

**DOI:** 10.3389/fonc.2022.1067312

**Published:** 2023-01-04

**Authors:** Bertrand Baussart, Alice Venier, Anne Jouinot, Gilles Reuter, Stephan Gaillard

**Affiliations:** ^1^ Department of Neurosurgery, La Pitié-Salpêtrière University Hospital, Assistance Publique-Hôpitaux de Paris, Paris, France; ^2^ Université Paris Cité, Institut Cochin, CNRS, INSERM, Paris, France; ^3^ Department of Neurosurgery, Neurocenter of Southern Switzerland, Lugano, Switzerland; ^4^ Department of Neurosurgery, Centre Hospitalier Universitaire (CHU) de Liège, Bat B35, Domaine Universitaire du Sart-Tilman, Liège, Belgium

**Keywords:** pituitary surgery, closure, skull base repair, endoscopy, strategy, nasoseptal flap

## Abstract

**Introduction:**

Effective strategies are required to ensure optimal management of the crucial closure step in endoscopic pituitary surgery. Many surgical techniques have been reported but no significant consensus has been defined.

**Methods:**

Between January 2006 and March 2022, 3015 adult patients with pituitary adenomas were operated on by a single expert neurosurgical team, using a mononostril endoscopic endonasal approach. Based of preoperative risk factors of and operative findings, a detailed closure strategy was used. Body mass index >40, sellar floor lysis, number of surgeries>2, large skull base destruction, prior radiotherapy were considered as preoperative risk factors for closure failure. All patients treated with an expanded endonasal approach were excluded.

**Results:**

Patients were mostly women (F/M ratio: 1.4) with a median age of 50 (range: 18 –89). Intraoperative CSF leak requiring specific surgical management was observed in 319/3015 (10.6%) of patients. If intraoperative leak occurred, patients with predictive risk factors were managed using a Foley balloon catheter in case of sellar floor lysis or BMI>40 and a multilayer repair strategy with a vascularized nasoseptal flap in other cases. Postoperative CSF leak occurred in 29/3015 (1%) of patients, while meningitis occurred in 24/3015 (0.8%) of patients. In patients with intraoperative leak, closure management failed in 11/319 (3.4%) of cases.

**Conclusion:**

Based on our significant 16-year experience, our surgical management is reliable and easy to follow. With a planned and stepwise strategy, the closure step can be optimized and tailored to each patient with a very low failure rate.

## Introduction

Pituitary adenomas account for 15% of all intracranial neoplasms, making them the third most common pathology (1). Pituitary adenomas, recently renamed as pituitary neuroendocrine tumors (PitNETs) in the new classifications (2–4) are usually benign tumors, with a broad spectrum of biological and pathological characteristics (5–7). Surgery represents the first-line treatment for most pituitary adenomas (corticotroph, somatotroph and non-functional), except for most prolactinomas which are currently treated with dopamine agonists (8).

The transsphenoidal approach is the gold standard of surgical route for pituitary surgery (9–11), while the transcranial approach is considered only as a second-line surgical option, in well-selected patients with rare tumors extending anteriorly in the subfrontal area, laterally in the temporal fossa or encompassing the vessels (12, 13). The microscopic technique, initially developed by Cushing and successively taken forward by Dott, Guiot and Hardy (14–16), was progressively abandoned in most centers of excellence, leading to the transition towards the endoscopic technique in the late 1990s (10, 17–19). Nowadays, the endoscopic endonasal transsphenoidal approach is the mainstay in many centers worldwide, given its advantages in terms of quality of vision, tumor resection, endocrine outcome, sinus-nasal morbidity and length of hospital stay (17, 20, 21).

Despite advances in closure techniques, postoperative CSF (cerebrospinal fluid) leak remains the most common complication of the endoscopic endonasal transsphenoidal approach, occurring in around 10% of patients and requiring a specific second surgery. The rate of CSF leak-related meningitis is observed in approximately 5% of patients, increasing the length of hospital stay and medical costs (22, 23). Thus, a reconstruction strategy of the skull-base defect should be anticipated during preoperative planning and an accurate analysis of preoperative risk factors associated with CSF leak is essential. In previous studies, many preoperative risk factors have been reported such as BMI (body mass index) > 30, multiple surgeries, tumor size, extension and invasiveness, prior treatment with radiotherapy (24–29). In all these patients, the closure strategy should be rigorously considered before the sellar surgical step, in order to limit avoidable reconstruction failures (24, 30) and to modulate it according to the flow of the CSF leak observed during surgery (31–34).

Today, several protocols to reduce postoperative CSF leak have been proposed (33–40) but there is no consensus on the best closure strategy after endoscopic pituitary surgery. Moreover, a significant heterogeneity in the outcomes was reported (41). From the analysis of the first 1000 patients operated on with a mononostril endoscopic endonasal transsellar approach, we reported in 2014 a postoperative CSK leak rate <1% (18). Based on our substantial additional experience, we developed a gradual closure strategy that integrates individual preoperative risks of postoperative CSF leak with the operative findings.

The objective of the present study is to analyze the results of our closure strategy from a consecutive cohort of 3015 patients with pituitary adenomas, operated on by the same two senior expert neurosurgeons (S.G, B.B). The philosophy of this major surgical step is emphasized, highlighting the need to plan the closure step before entering the operative room.

## Materials and methods

### Patients

This is a French observational cohort of 3015 consecutive adult patients with pituitary adenomas operated on between January 2006 and March 2022.

Inclusion criteria were: (1) diagnosis of adenoma confirmed on histological examination; (2) adenoma patients treated with a mononostril endoscopic endonasal transsphenoidal approach, as described in the “endoscopic endonasal transsphenoidal surgery” section; (3) patients eligible for surgery selected at a multidisciplinary meeting with an endocrinologist, a neurosurgeon and a radiologist; (4) dedicated pituitary MRI performed for each patient before surgery. Exclusion criteria were: (1) adenoma patients treated with an expanded endoscopic approach - such as transtuberculum or transplanum approach; (2) patients under 18 years of age.

Prior medical therapy with dopamine agonists or somatostatin analogues was not considered an exclusion criterion. All patients with prior medical therapy were included in this series.

### Predictive factors for closure failure

On the basis of previous studies (24, 25, 27–29, 42, 43), preoperative clinical and radiological assessment identified the following variables as preoperative risk factors for closure failure: severe obesity, number of surgeries > 2, focal sellar floor lysis, large skull base destruction due to invasive or giant pituitary adenomas and history of prior radiation therapy.

Intraoperative CSF leaks are often complex to treat in obese patients because of higher intracranial pressure (29). Furthermore, as previously published, the risk of symptomatic intracranial hypertension increases with increasing BMI. In patients with BMI>40, the risk of induced vision loss is well known (44). Based on this data, we have considered that patients with BMI>40 may be exposed to a higher risk of postoperative CSF leak due to increased intracranial pressure. Thus, this critical value was considered a risk factor of closure failure.

### Endoscopic endonasal transsphenoidal surgery

The same two senior neurosurgeons (S.G, B.B) operated on all patients via a mononostril endoscopic endonasal transsphenoidal approach, as recently described by the present team (20). The patient was placed in a semi-sitting position. Care was taken to avoid any compression points. During patient positioning, the right thigh was prepared for musculoaponeurotic graft whenever needed. The head was deflected back by 30° to prevent jugular compression. 0° and 30° optic endoscopes (Karl Storz) were used. After lateralization of the middle turbinate, the mucosa of the anterior part of the sphenoid bone was coagulated with a luxation of the septum. The sellar floor was opened and the dura incised. The adenoma was removed for pathological analysis using standard curettes, dissecting instruments, and suction.

**Figure 1:**
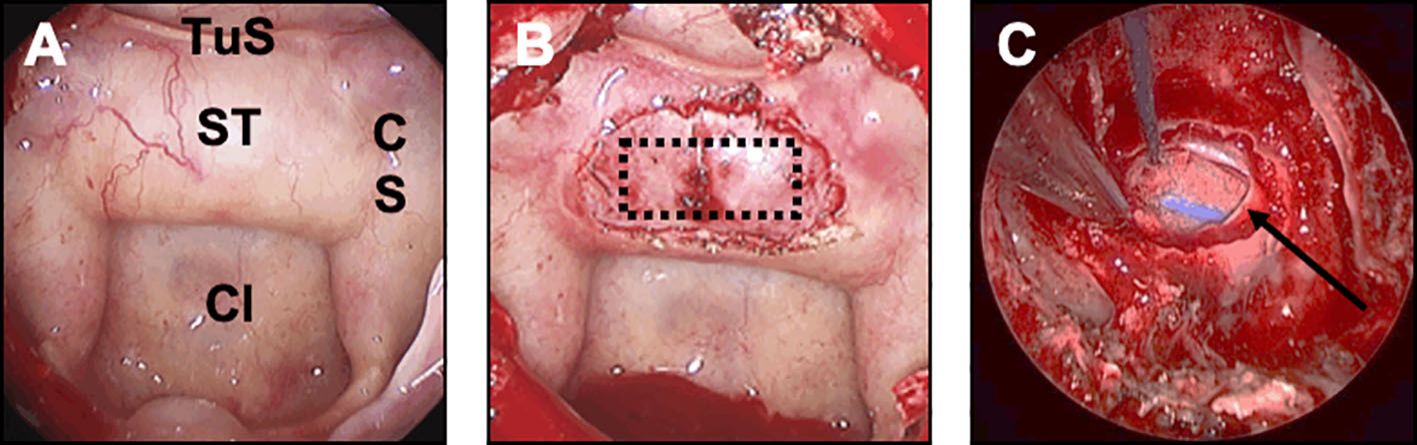
Preparation of the closure step during the endoscopic approach. This figure illustrates the concept that closure should be anticipated and planned for at each surgical step. **(A)** Sphenoid step. The main classic landmarks are identified: sella turcica (ST), tuberculum sellae (TuS), clivus (Cl), cavernous sinus (CS). **(B)** Sellar step. After opening the sellar floor, the dura has been carefully exposed and respected. The optimal rectangle shaped-dural opening is provided (dotted black rectangle). Note that the bone opening is oversized compared to the dural opening, in order to preserve the epidural space. **(C)** Sellar step after tumor resection. Note that the epidural space has been respected between the sellar floor and the dura mater (black arrow) for optimal closure.

The crucial closure step was anticipated during the approach: the main objective was to preserve an optimal epidural space when the sellar floor was opened and the tumor was removed, in order to reconstruct an optimal sellar floor during the closure step. The sellar floor was opened is such a way that the bone opening was larger than the dural opening, in order to preserve the epidural space (**Figure 1**).

### CSF leak evaluation

Once tumor was resected, the neurosurgeon had to determine the existence and intensity of a possible CSF leak. If a CSF leak was observed, the degree of CSF flow was assessed, as proposed by Esposito et al. (33). CSF leak was classified as follows: small leak without obvious diaphragm defect, defined as diaphragm oozing; low-flow leak with focal diaphragm defect; high-flow leak with large diaphragm or dural defect.

### Closure stage: General considerations

**Figure 2:**
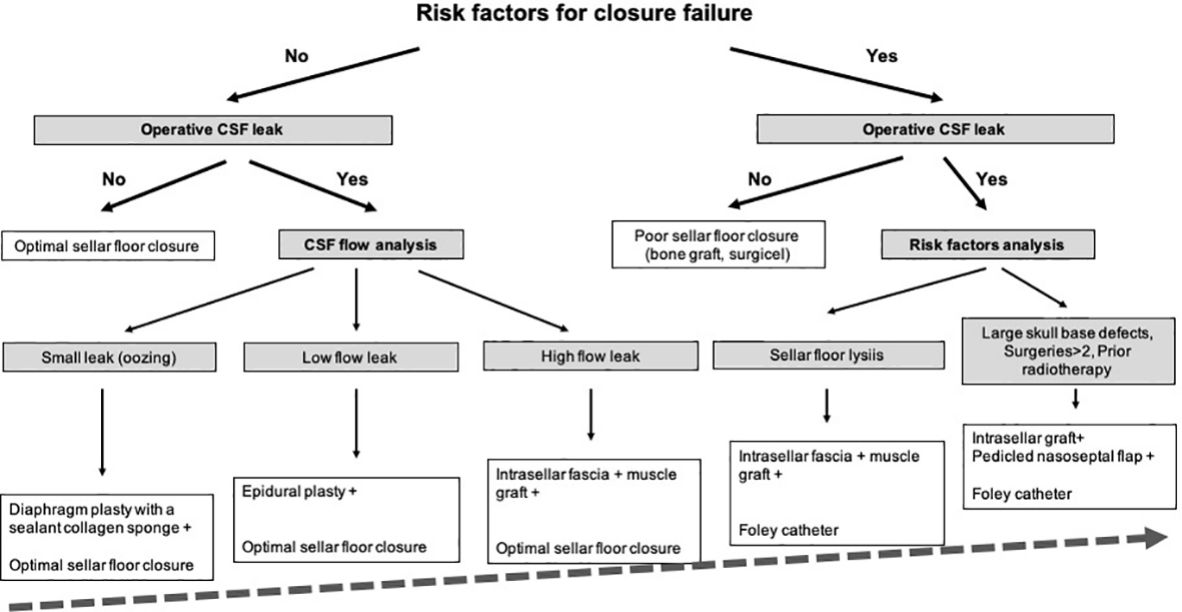
Closure strategy for pituitary surgery. Decision strategy.

The substantial experience gained since 2006 has allowed us to gradually establish a specific stepwise surgical strategy, based on a rigorous analysis of preoperative risk factors for closure failure and operative findings. The proposed closure strategy comes from a retrospective analysis of the authors’ experience gained during the study period. Our current strategy used since 2014 for graded closure strategy in pituitary surgery has been provided in **Figure 2**.

Indeed, some nuances have been added from our initial operative technique. Until 2014 (n=1397 patients), a 5-day external lumbar drainage could be decided as a second surgical option in combination with the intrasellar muscular graft, in rare patients with failure of closure management. Since 2014 (n=1618 patients), external lumbar drainage has been abandoned and replaced by the Foley Catheter technique: a saline-inflate Foley catheter was applied inside the sphenoid sinus in order to constitute an abutment against the intrasellar muscular graft (in case of sellar floor lysis or BMI>40) or against the double pedicled nasoseptal flap (in case of number of surgeries > 2, large skull base destruction, giant tumors or prior radiotherapy). This change essentially resulted from an objective to improve clinical tolerance, to reduce the risk of complications due to lumbar drainage and to treat more complex patients with more complex adenomas.

**Figure 3:**
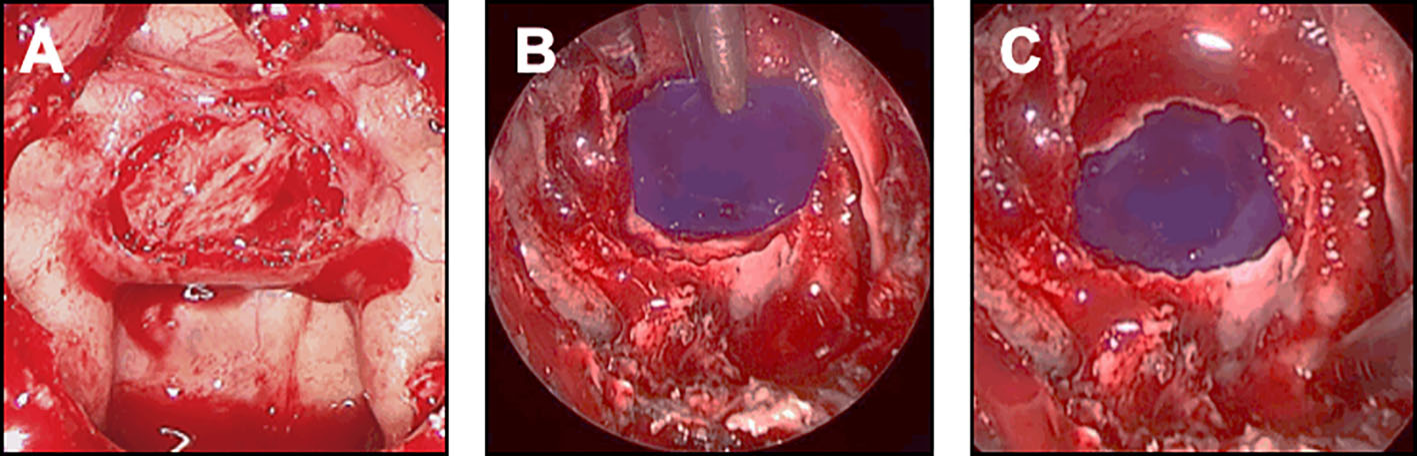
Closure strategy in patients with no CSF leak and no risk factor. For these patients, an optimal sellar floor closure should be achieved. **(A)** Sellar reconstruction with a bone graft. The piece of bone has been removed from the sphenoid rostrum or from a sphenoid septation, accurately designed and positioned in the previously prepared epidural space. **(B, C)** Sellar reconstruction with a synthetic polydioxanone (PDS) plate. **(B)** A synthetic polydioxanone (PDS) plate (in blue) has been shaped according to the sellar floor opening and introduced in the sphenoid sinus. The positioning always starts with the introduction of the two lower edges, the plate being held by a surgical forceps. **(C)** The two upper edges have been embedded so that the entire PDS plate was positioned in the epidural space.

If possible, no material was placed in the intrasellar compartment, so as not to interfere with the interpretation of the postoperative MRI and not to impact on tumor resection in any subsequent surgery. In the absence of CSK leak, an optimal standard bone sellar floor reconstruction was achieved, using an autologous bone from a sphenoid septation designed in a quadrangular shape and positioned by four corners in the epidural space (**Figure 3**). In case of patients with strong risk factors of postoperative CSF leak (large skull base destruction due to invasive or giant pituitary adenomas, number of surgeries >2, history of prior radiotherapy), a multilayer reconstruction strategy was decided, using the double pedicled nasoseptal flap, as previously reported (45, 46).

### Closure strategy

#### i. Patients with no risk factors of closure failure

In the absence of CSK leak, optimal bone sellar floor reconstruction was performed, as detailed above. In case of intraoperative CSF leak, the choice of the closure depended on the intensity of flow and the location of the leak.

**Figure 4:**
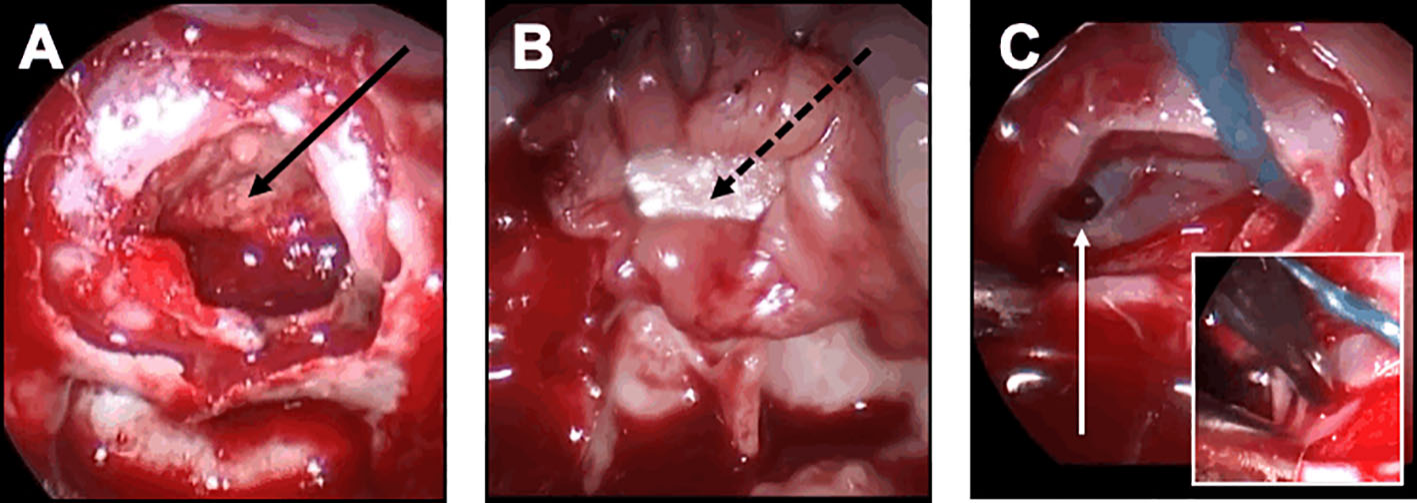
Closure strategy in patients with CSF leak and no risk factor. For these patients, the sella floor should be reconstructed and biological glue should be applied after the sealing has been achieved. **(A, B)** Minimal and diffuse flow diaphragm leak. **(A)** A sealant collagen sponge coated with the human coagulation factors fibrinogen and thrombin (black arrow) has been designed, positioned in the intrasellar compartment, deployed, centered on the dural defect and applied against the diaphragm with the help of a forceps holding a cottonoid and a suction tube. **(B)** An epidural duraplasty has been performed, using the mucosa of the right middle turbinate. After the mucosa has been embedded in the epidural space with a bone graft (black dotted arrow), the mucosa acts as a seal. **(C)** Focal low-flow leak. A unique focal defect is visualized on the right side of the diaphragm (white arrow). The sealing is achieved after coagulation of the dural defect using a bipolar forceps (insert).

In case of small leak due to diaphragm oozing, a collagen sponge coated with the human coagulation factors fibrinogen and thrombin (TachoSil®) was used. The sealant matrix was positioned in the intrasellar compartment, deployed, centered on the dural defect and applied against the diaphragm with the help of forceps holding a cottonoid and a suction tube (**Figure 4A**). The sellar floor was reconstructed and some biological glue was applied in the sphenoid sinus.

In case of focal low-flow leak, an epidural duraplasty was performed, using a dural substitute. After a right middle turbinectomy was performed, the mucosa of the turbinate was removed away from the bone. The mucosal graft from the middle turbinate was positioned in the epidural space, with the support of a bone graft or a PDS plate embedded in an epidural fashion (**Figures 4A, B**). With this technique, the mucosa should act as a seal. As previously described, biological glue was applied. In rare cases of focal diaphragm defect with a distended diaphragm bulging into the intrasellar space, watertight closure could be achieved by coagulating the edges of the transdiaphragmatic orifice with bipolar forceps (**Figure 4C**).

In case of diffuse of high-flow leak, an intrasellar packing technique was decided. Fascia and muscle grafts were taken from the right thigh. The fascia was introduced into the sella and applied superiorly to cover the entire defect, in order to recreate a new diaphragm. The muscle graft was then positioned within the intrasellar compartment. As previously described, the sellar floor was reconstructed with bone graft or PDS plate and biological glue was applied.

#### ii. Patients with risk factors of closure failure

##### BMI>40 with intact sellar floor, no intraoperative leak

A standard closure was performed, as previously described.

##### Focal sellar floor lysis and BMI<40, no intraoperative leak

A poor sellar closure was usually achieved, using a bone graft or PDS plate positioned by only two or three corners in the epidural space

##### Focal sellar floor lysis and BMI>40, no intraoperative leak

An additional Foley catheter technique was decided in order to avoid the migration of the poor sellar reconstruction. A two-way Foley balloon catheter technique was used: the balloon stent was positioned inside the sphenoid sinus against the sella turcica to reinforce the reconstruction and counter the effects of graft migration. Usually, we used a Foley urinary catheter from 8 to 12 French filled up with saline solution, inflating it to be in contact with the graft. The catheter was left in place for 4 to 5 days.

##### Focal sellar floor lysis and/or BMI>40, intraoperative leak

All patients were treated with an intrasellar fascia and muscle graft, combined with an additional Foley balloon catheter. Biological glue was always applied. Repeated lumbar punctures were performed on day 1 and day 2 after surgery

##### Large skull base destruction and/or number of surgeries >2 and/or prior radiotherapy

**Figure 5:**
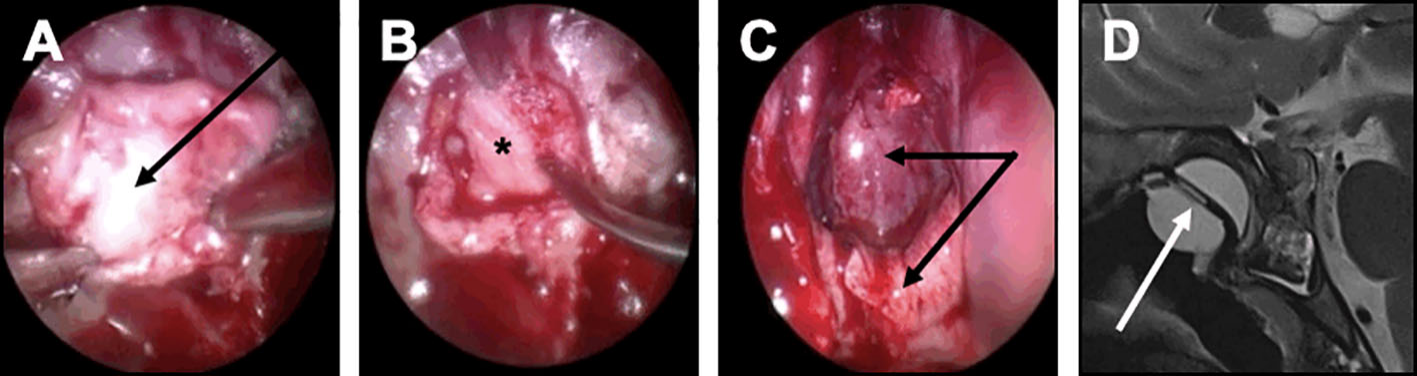
Closure strategy in patients with a risk factor. For these patients, a Foley urinary catheter is usually necessary and more complex multilayer closure strategies using a pedicled mucosal nasoseptal flap should be discussed. **(A-D)** Complex multilayer closure strategy for a patient with a recurrent pituitary adenoma treated with multiple surgery and radiotherapy. **(A)** After a muscle graft has been introduced in the intrasellar compartment, an autologous fascia lata graft has been positioned anterior to the sella turcica (black arrow). **(B)** After the fascia lata has been embedded in the epidural space with a bone graft (black asterisk), the primary sealing is obtained. **(C)** A vascularized mucosal nasoseptal flap is positioned and deployed in the sphenoid sinus (double black arrow), maintained using a Foley urinary catheter for 5 days. **(D)** Postoperative MRI showing the multilayer reconstruction and the Foley catheter (white arrow).

In complex cases of patients with strong risk factors of closure failure, the following strategy was decided, regardless the occurrence of an intraoperative leak: a multilayer repair strategy was chosen with combined intrasellar fascia and muscle grafts, optimal epidural closure if possible, and double pedicled mucosal nasoseptal flap (**Figure 5**). Each left and right mucosal flap was elevated from each side of the nasal bone septum and pedicled on the sphenopalatine artery. The pedicled flap was applied against the sella turcica and held in place with a Foley catheter for 5 days. Biological glue was always applied. Repeated lumbar punctures were performed on day 1 and day 2 after surgery. This multilayer strategy was also decided in case of patients with BMI>40.

### Closure evaluation

Closure was considered as achieved if no postoperative CSF leak was observed 2 months after surgery. Failure of closure management was diagnosed when a postoperative CSF leak occurred despite the closure strategy applied because of an intraoperative CSF leak during the first procedure, requiring a second surgery.

### Data collection

The following variables were collected for each patient before, during, and after surgery.

- Before surgery: age; sex; preoperative risk factors for closure failure.
- During surgery: intraoperative CSF leak; flow intensity and location of intraoperative CSF; type of closure strategy.
- After surgery: postoperative CSF leak; type of closure strategy; meningitis with bacteriological analysis if available.

### Statistical analysis

Statistical analysis was performed using R statistical software (version 3.6.3). Descriptive statistics used median (range) for quantitative variables and raw numbers (%) for categorical variables.

## Results

### Characteristics of patients selected for surgery

The characteristics of 3015 patients operated on for an adenoma are provided in **Table 1**. Patients were mostly women (F/M ratio: 1.4), with a median age of 50 (range: 18 to 89). Hormone excess was observed in 1970/3015 (65.3%) of patients. Corticotroph adenomas with Cushing’s disease, somatotroph adenomas with acromegaly, lactotroph adenoma, thyrotroph adenoma and non-secreting adenomas were diagnosed in 822/3015 (27.3%), 768/3015 (25.5%), 331/3015 (11%), 49/3015 (1.6%) and 1045/3015 (34.6%) of patients respectively.

**Table 1:** Characteristics of 3015 adenoma patients treated with a mononostril endoscopic endonasal transsellar approach.

	Patients
**Age**, years	50 (18-89)
**Sex ratio (F/M)**	1.4
	
**Type of secretion**	
Cushing’s disease	822/3015 (27.3%)
Acromegaly	768/3015 (25.5%)
Lactotroph adenoma	331/3015 (11%)
Thyrotroph adenoma	49/3015 (1.6%)
Non-secreting adenoma	1045/3015 (34.6%)
	
**Intraoperative CSF leak requiring a specific surgical management**	**319/3015 (10.6%)**
**Intraoperative CSF leak with no risk factor of closure failure**	**258/319 (80.9%)**
Diaphragm oozing treated with collagen sponge	32/258 (12.4%)
Focal low-flow leak treated	83/258 (32.2%)
treated by focal coagulation	6/83 (7.2%)
treated with an extrasellar epidural graft	77/83 (92.8%)
Diffuse high-flow leak treated with intrasellar fascia and muscle grafts	143/258 (55.4%)
treated with intrasellar fascia and muscle grafts	142/143 (99.3%)
treated with epidural graft	1/143 (0.7%)
	
**Intraoperative CSF leak with a least one risk factor of closure failure**	**61/319 (19.1%)**
BMI>40 and/or Sellar floor lysis treated with a Foley balloon Catheter	39/61 (63.9%)
BMI>40 and/or Sellar floor lysis treated with collagen sponge, epidural or muscle graft	6/61 (9.8%)
Large skull base destruction and/or prior radiotherapy and/or surgeries >2 treated with a mucosal nasoseptal flap	16/61 (26.3%)
	
**Postoperative CSF leak requiring a second surgery**	**29/3015 (1%)**
Patients with no intraoperative CSF leak	18/29 (62.1%)
Patients with intraoperative CSF leak	11/29 (37.9%)
**Rate of failed closure management**	**11/319 (3.4%)**
	
**Meningitis requiring antibiotic therapy**	**24/3015 (0.8%)**
Aseptic meningitis	14/24 (58.3%)
Septic meningitis	10/24 (41.7%)
	
Patients with intraoperative CSF leak	19/24 (79.2%)
Patients with no intraoperative CSF leak	5/24 (20.8%)
	
Patients with postoperative CSF leak	5/24 (20.8%)
Patients with no postoperative CSF leak	19/24 (79.2%)

Quantitative variables are expressed in median (range); qualitative variables are expressed in absolute numbers (proportion).

### Intraoperative CSF leak

Intraoperative CSF leak requiring a specific surgical management was observed in 319/3015 (10.6%) of patients.

No preoperative risk factor of closure failure was noted in 258/319 (80.9%) of these patients. Diaphragm oozing was noted in 32/258 (12.4%) of patients and was treated with collagen sponge (TachoSil® patch). Focal low-flow leak was noted in 83/258 (32.2%) of patients, mainly treated with epidural graft and more rarely with bipolar coagulation. Diffuse high-flow leak was observed in 143/258 (55.4%) of patients and was treated with intrasellar fascia and muscle graft technique in the vast majority of cases.

Preoperative factors of closure failure were observed in 61/319 (19.1%) of patients. Considering BMI>40 and/or sellar floor lysis, 39/61 (63.9%) of patients were treated with the Foley-catheter technique, while 6/61 (9.8%) were treated using collagen sponge, epidural graft or muscle graft. The 16/61(26.3%) patients with large skull base destruction and/or prior radiation therapy and/or number of surgeries >2 were treated with a multilayer repair strategy with nasoseptal flaps.

### Postoperative CSF leak

Postoperative CSF leak requiring a second surgery was observed in 29/3015 (1%) of patients.

Among these patients, 18/29 (62.1%) had no intraoperative CSF leak. No risk factors for closure failure were noted in these 18 patients. In 2 patients, a postoperative leak occurred 5 and 6 days after surgery, in the context of nose blowing.

In contrast, 11/29 (37.9%) of patients had a well-identified intraoperative leak requiring a dedicated closure technique, as detailed in **Table 2**. The majority of these 11 patients had preoperative risk factors (n=7/11, 63.6%), mostly with BMI >40. Diaphragm oozing, low-flow leak and high-flow were identified in 2/11 (18.2%), 4/11 (36.4%) and 5/11 (45.4%) of patients respectively. All these 11 patients were reoperated on, using an intrasellar fascia and muscle graft combined with external lumbar drainage in 3 patients (before 2014) and Foley-catheter or mucosal flap in 2 patients (after 2014). A permanent lumboperitoneal shunt was needed in 2 patients. No difference in postoperative leak rate was observed after the change of our surgical strategy in 2014 (5 patients identified before 2014 and 6 patients identified after 2014). Considering the 11 patients with both intra and postoperative leak and all 319 patients with intraoperative leak, the rate of failed closure was 3.4%. The closure failure was mainly due to poor analysis of intraoperative CSF flow.

**Table 2:** Characteristics of the 11 patients with failed closure management despite a well-identified intraoperative CSF leak.

Patient	Age	Sex	Date of Surgery	Nb of surgeries	BMI>40	Lysis of sellar floor	Skull base destruction	Giant tumor	Prior radiotherapy	CSF Flow	First closure strategy	Meningitis	Second closure strategy	Hypothesis forclosure failure
1	57	M	2008	1	1	1	1	1	0	Oozing	Surgicel	0	Intrasellar graft	Poor material selection
2	42	M	2009	2	0	1	1	1	0	High	Intrasellar graft	1	Intrasellar graft + External lumbar drain Lumboperitoneal shunt	Poor graft design
3	43	M	2008	2	0	1	1	1	1	High	Intrasellar graft	0	Intrasellar graft + External lumbar drain Lumboperitoneal shunt	Poor graft design
4	68	M	2012	1	0	0	0	0	0	High	Epidural graft	0	Intrasellar graft + External lumbar drain	Poor choice of closure strategy
5	79	F	2013	1	0	0	0	0	0	Oozing	Tachosil	0	Intrasellar graft	Poor analysis of CSF flow
6	37	F	2015	1	1	0	0	0	0	Low	Epidural graft	0	Intrasellar graft	Poor analysis of CSF flow
7	51	F	2015	2	1	1	0	0	0	High	Intrasellar graft	0	Intrasellar graft + Foley catheter	Poor evaluation of predictive factor
8	43	F	2018	1	1	0	0	0	0	Low	Epidural graft	0	Intrasellar graft	Poor analysis of CSF flow
9	22	M	2019	2	0	0	0	0	0	Low	Epidural graft	1	Intrasellar graft	Poor analysis of CSF flow
10	59	M	2019	1	0	0	0	0	0	Low	Epidural graft	0	Intrasellar graft	Poor analysis of CSF flow
11	27	M	2019	1	1	1	1	1	0	High	Intrasellar graft	0	Intrasellar graft+ Mucosal flap	Poor evaluation of predictive factor

### Postoperative meningitis

Meningitis occurred in 24/3015 (0.8%) of patients. Aseptic meningitis, diagnosed on the basis of fever, clinical symptoms and repeated CSF examinations (cells increase, low glucose, high protein), was found in 14/24 (58.3%) of patients, while septic meningitis with a well-documented bacteriological analysis was reported in 10/24 (41.7%) of patients. CSF examination revealed Gram-positive bacteria in 6 patients (Coagulase-negative staphylococci in 4 patients and Staphyloccocus aureus in 2 patients) and Gram-negative in 4 patients (Klebsiella pneumonia in 3 patients and Escherichia coli in 1 patient). All patients were treated with antibiotic therapy and had a favorable outcome.

Intraoperative leak was observed in 19/24 (79.2%) of patients with meningitis, while postoperative leak was observed in 5/24 (20.8%) of patients.

## Discussion

This paper focuses on the evaluation of the closure step in pituitary surgery, based on a large series of 3015 adenoma patients treated with a mononostril endoscopic endonasal transsellar approach by the same expert surgical team. In PitNETs, invasion of the basal dura, cavernous sinus and/or diaphragm is encountered in up to 35% of patients (2), explaining the substantial occurrence of intraoperative leak requiring reliable watertight closure techniques. Initially the endoscopic technique was associated with high reported rates of postoperative CSF leak, ranging from 30 to 40% (47). With advances in endoscopic pituitary surgery and the development of new reconstruction techniques, optimized lower rates <10 % are currently reported (24, 40, 48–50). The present study demonstrates that our stepwise strategy is safe, reliable and effective. Applying our decision strategy, our rates of postoperative CSF leak and meningitis were 1% and 0.8% respectively, which compares favorably with the best rates previously reported (34, 51).

In pituitary surgery, the crucial step of closure should be anticipated. The neurosurgeon should keep in mind that the closure step already begins during the approach. Even if no intraoperative leak occurs, the overall concept is to preserve a good epidural space during the approach and to recreate a sellar floor whenever possible. Indeed, sellar floor closure is useful to identify bony landmarks in the event of a later second surgery and may limit the risk of postoperative CSF leak related to a secondary stall of the diaphragm during sneezing or blowing. During the approach, the sellar floor is opened is such a way that the bone opening is larger than the dural opening, in order to preserve the epidural space (34, 38, 52). When possible, an autologous bone graft is removed and preserved from the sphenoidal rostrum and/or one of a sphenoid sinus septation. If no bone graft is available, a synthetic resorbable polydioxanone (PDS) plate can be used with similar shaping and positioning (53). We choose to use the PDS material because of its stiffness, which is close to that of bone. We recommend the use of a resorbable sellar floor substitute to limit the risk of infectious complications.

In case of intraoperative CSF leak, our patients were divided into two groups on the basis of selected preoperative risk factors of closure failure (28, 29, 43). A specific gradual closure strategy was planned accordingly. Interestingly, other preoperative risk factors of closure failure have been reported recently by expert teams, such as suprasellar extension, chronic respiratory disease, type of sellar barrier, fibrous consistency, dumbbell-shape or lobulated asymmetrical configuration (42). A comprehensive analysis of all these factors should allow surgical management to be tailored to the individual patient.

In patients with no risk factor, the objective is to achieve a watertight closure and reconstruct the sellar floor. Different materials have been proposed for the reconstruction of skull-base defects. Autologous materials have been proposed, such as mucosal grafts from middle turbinate, fat grafts from abdominal region, muscle graft from lateral thigh, fascia grafts from fascia lata, lateral thigh or temporal muscle (31, 32, 48, 53–58). Heterologous biologic dural substitutes have been used, such as equine pericardium sheet (59) or human-derived acellular dermal matrix (60). Heterologous synthetic dural substitutes have also been proposed, such as polyester-silicone (61), resorbable polyglactin acid sheet (62), polytetrafluoroethylene (63) or collagen matrix (64, 65). At the end of the closure procedure, fibrin glue should is usually applied inside the sphenoidal cavity to fill the dead spaces (66–68). In our strategy, the key objective was to minimize the risk of postoperative leak while minimizing the proportion of patients treated by packing the sella or sphenoid sinus. Indeed, packing the sellar area with additional fibrous scar tissue may impact on postoperative MRI analysis and alter the quality of surgical landmarks in case of repeated surgeries. The choice of the closure technique depends on the flow intensity and on the leak location, as proposed by Conger et al. (34). In case of minimal diaphragm oozing, a simple treatment with a collagen sponge can be chosen (64, 66, 69). Collagen sponge has the great advantage to be easy to use. However, if the CSF is underestimated, this closure technique will be insufficient and a postoperative CSF leak will occur, requiring a second surgery. We therefore recommend spending a lot of time analyzing the intensity of CSF flow and adopting a safer alternative reconstruction strategy when in doubt. Focal low-flow intraoperative leak requires a stronger closure strategy: although an adequate epidural graft is more complex to perform, this strategy is well suited in this case, with a high rate of watertight closure without intrasellar packing. An epidural duraplasty can be performed, using a dural substitute, such as mucosa from a middle turbinate or fascia lata, held in place by a rigid buttress, as previously reported under different names, such as the gasket seal technique (34, 52, 70). However, the surgeon should be aware that if part of this duraplasty is positioned inside the intradural intrasellar space, the strategy will not prevent a postoperative leak. In our experience, the graft will paradoxically have a “gutter effect” with an increased risk of postoperative leak. Of note, in rare cases of focal diaphragm defect with a distended diaphragm bulging into the intrasellar space, a watertight closure can be elegantly obtained by coagulating the edges of the transdiaphragmatic orifice with bipolar forceps. In case of high-flow leak, the objective is to achieve a strong and persistent watertight closure with an intrasellar packing. Some authors recommend to use intrasellar fat and glue (33, 57, 71–75) with low rates of postoperative leak. In our experience, we prefer to use a fascia and muscle graft to achieve a two-layer intrasellar repair: the fascia is introduced into the sella and applied superiorly to cover the entire defect, in order to recreate a new diaphragm; the muscle graft is then positioned in the intrasellar compartment. Special care must be taken in the design of the muscle graft to avoid significant mass effect with compression of the optic chiasma.

In patients with risk factors, more complex strategies should be decided. In case of sellar floor lysis, bone may be missing, which is why no solid epidural buttress can be performed. Solutions with a buttress positioned within the sphenoid sinus have been developed. Some authors proposed a fat buttress to pack the sphenoid sinus (33, 57, 76, 77). The disadvantage of this technique is that the counterpressure is not applied in a targeted manner against the sellar floor and the fibrous scarring may complicate the surgical approach if further intervention is required. For these reasons, we prefer to use a Foley balloon catheter inflated within the sphenoid sinus for a few days, as previously described (78). Thus, the transient buttress is directly applied against the sella floor until the intrasellar muscle graft can no longer move. In more complex patients with giant tumors and large dural defects, a multilayer strategy with mucosal flap is usually recommended. Vascularized grafts have been a surgical revolution in preventing postoperative CSF leak, especially in patients with risk factors (24, 25, 48, 49, 79, 80). Different types of vascularized grafts have been described: unilateral or bilateral mucosal flaps originating from nasal septum or inferior turbinate; pericranium or temporal fascia flaps (45, 46, 48, 53, 54, 56, 81, 82). The choice of using vascularized flaps involves more complex skull base reconstruction techniques with multilayered closure and should balance the risk of postoperative CSF leak versus the morbidity of the flap itself (30, 83–85).

All 29 patients with postoperative CSF leak were reoperated with a dedicated upgraded closure strategy. Our management has evolved over the time. Indeed, the use of external lumbar drainage in these patients is associated with a higher risk of complication (86, 87). Of note, as previously reported, there is a lack of statistically significant improvement between patients with lumbar drains and patients with no lumbar drains and graded reconstruction strategies without lumbar drainage have been proposed (39, 88–90). Today, external lumbar drainage has been abandoned for the overwhelming majority of our patients treated with simple transsellar approach.

Postoperative CSF leak occurred in 18 patients with no intraoperative CSF leak identified. In 2 patients, the postoperative leak was obviously caused by inappropriate postoperative nose blowing. In 16 patients, the complication may have been due to intraoperative surgical misinterpretation, related to unnoticed diaphragm oozing.

Despite of our closure strategy, surgery failed in 11 patients with a well-identified intraoperative CSF leak, leading to an overall closure failure rate of 3.4%. Interestingly, most of the patients were treated before 2018, suggesting that this type of complication may decrease with endoscopic surgical experience, as previously proposed (91). Firstly, lack of experience with endoscopic techniques may have led to poor material selection or poor graft design, as previously reported (92–94). Secondly, the majority of these patients had at least one risk factor of closure failure. Thus, surgical failure was also explained by underestimated risk factors, such as severe obesity; BMI>40 is associated with increased intracranial pressure (44), which may affect the quality of closure (29). In patients with BMI>40 and focal sellar floor lysis, we now recommend the use of an additional Foley-catheter combined with repeated lumbar punctures, even if no CSF occurred during surgery. Closure failure was finally caused by poor operative conditions reducing the quality of vision and impacting on the analysis of CSF flow.

Our rate of postoperative meningitis was 0.8%, with a majority of aseptic meningitis. This result is in accordance with previous studies from pituitary centers of excellence (51, 95). In patients with no identified bacteria, antibiotic treatment was indicated on the basis of combined neurological symptoms (fever, meningeal syndrome) and analysis of repeated CSF examinations (cells increase, low glucose, high protein). These patients may have been overtreated. Nevertheless, all symptoms improved immediately after treatment was introduced, suggesting that a small bacterial inoculum was still present. In patients with septic meningitis, drug-sensitive Gram-positive positive organisms were predominant. All patients were treated by antibiotic therapy, with favorable outcome. Our results confirmed the data published by Jin et al. in a large retrospective study of 3242 patients (51). Interestingly, the vast majority of our patients (79.2%) with meningitis had a well-identified intraoperative leak, whereas a postoperative CSF leak was observed in only 20.8% of these patients. This result may suggest that the duration of CSF leak is not a strong predictor of postoperative meningitis. More data are needed to confirm this finding.

The main strength of this major study was the consecutive inclusion of all cases of adenoma patients treated with an endoscopic endonasal transsellar approach, reaching the substantial number of 3015 patients. Conversely, the inclusion of patients operated on by a single surgical team may be considered as a limitation. However, as mainly reported, the high expertise of surgical centers is essential (92, 96, 97). In this series, all patients were operated on by two experienced neurosurgeons treating >200 adenomas per year, after surgical indication was validated in a multidisciplinary meeting. If the expertise is not guaranteed, it can be hypothesized that outcome would become less favorable. Thus, at the beginning of the learning curve or if there is any doubt about a high-flow leak, safety should be paramount and intrasellar packing should be chosen. Due to the length of the study, our list of predictive factors is not exhaustive, which is a limitation of the present work. The main objective was to propose a graded closure strategy with excellent efficacy, based on our significant experience. Further studies with stronger decision-making paradigms, including more predictive factors, are needed in the future.

In conclusion, in pituitary surgery, the closure step should not be underestimated. By using a rigorous strategy, the postoperative leak rate can be reduced to 1% of patients. This crucial step must be planned before surgery and gently prepared during the surgical approach.

## Data availability statement

The raw data supporting the conclusions of this article will be made available by the authors, without undue reservation.

## Ethics statement

In accordance with the French legislation, patient consent was not needed for this retrospective non-interventional study evaluating a routine care. An agreement was obtained after ethical acceptance of the study by the General Register of the Assistance Publique des Hôpitaux de Paris - Sorbonne University (registered under “CLOSPIT” Study, N°: 202211210146).

## Author contributions

BB, AV, and SG contributed to conception and design of the study. BB, AJ, and AV organized the database. BB and AJ performed the statistical analysis. AV wrote the first draft of the manuscript. BB, AV, AJ, GR, and SG wrote sections of the manuscript. BB, AJ, GR, and SG contributed to interpretation of data for work. All authors contributed to manuscript revision, read, and approved the submitted version
